# Smart Nanoformulations for Brain Cancer Theranostics: Challenges and Promises

**DOI:** 10.3390/cancers14215389

**Published:** 2022-11-01

**Authors:** Faraz Ahmad, Ressin Varghese, Subhrajita Panda, Siva Ramamoorthy, Mohammad Y. Areeshi, Sharmila Fagoonee, Shafiul Haque

**Affiliations:** 1 Department of Biotechnology, School of Bio Sciences and Technology (SBST), Vellore Institute of Technology, Vellore 632014, India; 2 Research and Scientific Studies Unit, College of Nursing and Allied Health Sciences, Jazan University, Jazan 45142, Saudi Arabia; 3 Institute of Biostructure and Bioimaging (CNR), Molecular Biotechnology Center, 10126 Turin, Italy

**Keywords:** gliomas, theranostics, bioengineering, electro-magnetic nanoparticles, focused ultrasound, exosomes

## Abstract

**Simple Summary:**

Brain metastases are amongst the most invasive and lethal cancers. Yet, effective methods for their diagnosis and therapy have remained elusive. While several factors are responsible for this, the primary hurdle has been the absence of an appropriate delivery strategy for the diagnostic/therapeutic agents to the brain. Fortunately, the advent of nanobiotechnology and the advances in bioengineering techniques have provided some hope to circumvent this hurdle. The aim of this review is to evaluate the suitability of novel smart nanobiotechnological approaches in brain cancer therapy. We discuss several state-of-the-art strategies for specific delivery of drugs to brain cancer sites with minimal side effects. We further delineate several classes of nanoplatforms that have generated tremendous interest among contemporary scientists as potential agents for brain cancer therapy. In doing so, the authors hope that the review will serve as a platform for further studies for the discovery of brain cancer strategies.

**Abstract:**

Despite their low prevalence, brain tumors are among the most lethal cancers. They are extremely difficult to diagnose, monitor and treat. Conventional anti-cancer strategies such as radio- and chemotherapy have largely failed, and to date, the development of even a single effective therapeutic strategy against central nervous system (CNS) tumors has remained elusive. There are several factors responsible for this. Brain cancers are a heterogeneous group of diseases with variable origins, biochemical properties and degrees of invasiveness. High-grade gliomas are amongst the most metastatic and invasive cancers, which is another reason for therapeutic failure in their case. Moreover, crossing the blood brain and the blood brain tumor barriers has been a significant hindrance in the development of efficient CNS therapeutics. Cancer nanomedicine, which encompasses the application of nanotechnology for diagnosis, monitoring and therapy of cancers, is a rapidly evolving field of translational medicine. Nanoformulations, because of their extreme versatility and manipulative potential, are emerging candidates for tumor targeting, penetration and treatment in the brain. Moreover, suitable nanocarriers can be commissioned for theranostics, a combinatorial personalized approach for simultaneous imaging and therapy. This review first details the recent advances in novel bioengineering techniques that provide promising avenues for circumventing the hurdles of delivering the diagnostic/therapeutic agent to the CNS. The authors then describe in detail the tremendous potential of utilizing nanotechnology, particularly nano-theranostics for brain cancer imaging and therapy, and outline the different categories of recently developed next-generation smart nanoformulations that have exceptional potential for making a breakthrough in clinical neuro-oncology therapeutics.

## 1. Introduction

Brain disorders are among the most prevalent of disorders, yet strategies for their diagnosis and treatment remain mediocre at best. The global surge in drug development for neuropathologies has grown rapidly with the increase in the elderly population and the number of confirmed clinical cases. Intriguingly, drug development for diseases of the central nervous system (CNS) has a very poor success rate. The failures of clinical trials of the potential drugs/agents are largely attributed to the complexity of the brain and the multifactorial nature of its disorders, undesirable side effects and the highly selective nature of the blood brain barrier (BBB). The latter has in particular been a major hindrance, since only low molecular weight lipophilic molecules can effectively cross the BBB, rendering the vast majority of potentially neuroprotective molecules inefficient [1].

Among the diverse cancer types, brain cancers have one of the lowest incidences; however, they are amongst the most invasive and with the highest mortality rates [2]. In addition to the excessive rates of recurrence and low survival rates, hindrances to common anti-cancer drugs make brain cancers one of the most dreadful cancers [3]. In spite of this, CNS tumors are also amongst the least studied, and relatively little is known about their pathogenesis, and significant challenges are often encountered during their correct clinical diagnosis. Brain tumors can elicit in people of all age groups, but are more prevalent in children and older adults [4]. As such, they are a diverse group of cancers with varied ranges of malignancies and distinct pathological and biological characteristics. CNS tumors have been classified by the World Health Organization based upon their origin, molecular and histological parameters and their proliferative potential and likelihood of dissemination [5,6]. Broadly, they are classified as primary (originating from brain cells) or the more prevalent secondary or metastatic (originating elsewhere in the periphery and migrating to the CNS) brain tumors. All secondary brain tumors are malignant, but primary brain tumors can be benign or malignant. The primary malignant brain tumors, most of them originating from glial cells (hence called gliomas), constitute the major type of CNS tumors and elicit rapid progression and often fatal outcomes within months of diagnosis [7,8]. Moreover, they are extremely heterogeneous in nature, and among them grade IV malignant glioma or glioblastoma multiforme (GBM) shows the highest malignancy and invasiveness [6,8].

The standard clinical therapy in malignant brain tumors is notoriously complicated and involves maximal surgical resection in combination with high-dose radiotherapy and chemotherapy; however, the median survival time and recurrence time is still very small [9,10]. In addition to the highly invasive and recurring nature of brain cancers, the major challenges in their treatment include resistance to common chemo- and radio-therapies and side-effects (toxicity to healthy cells) [11]. Chemotherapeutics that have improved the prognosis of many other cancers have largely failed against brain tumors, primarily owing to the inability to penetrate the blood brain and blood brain tumor barriers (BBTB) [12,13]. Several other properties of CNS cancers have contributed to the failures of conventional therapeutics and elusiveness of development of an effective therapy, such as their cellular and molecular heterogeneity, immunosuppressive nature and susceptibility to genetic/epigenetic influences [2,9]. Overall, the failures in clinical neuro-oncology call both for a better understanding of the molecular and cellular mechanisms of pathogenesis of brain tumors (and their heterogeneity/plasticity) as well as the design of innovative and insightful treatment/monitoring approaches. Given the heterogeneity of brain cancers, it is clear that the way forward in clinical neuro-oncology is to develop combinatorial theranostic approaches. Strategies for the simultaneous imaging and treatment of brain cancers may have several advantages. They provide a suitable platform for monitoring drug accumulation at the intended site and the progression/regression and recurrence of the cancer, allowing evaluation of alternative strategies for non-responders. Theranostic strategies are particularly beneficial in an intraoperative setting, wherein clear demarcations between cancerous and healthy tissue may allow for accurate surgical resection [14,15].

The ever-evolving field of nanotechnology has opened new avenues for the study and regulation of biological systems at the nanoscale level by mimicking or manipulating endogenous cellular mechanisms. In particular, because of their versatilities and unique potential for manipulation/functionalization, smart nanomedicines hold tremendous promise for theranostic diagnosis and management of brain tumors [16,17]. The first and obvious advantage of using nanoparticles (NPs) is their size, which confers the potential ability to cross the BBB/BBTB via the enhanced permeability and retention effect (EPR; see Section 2.3) [18,19]. Moreover, nanoengineering-mediated alterations in their physico-chemical characteristics can optimize the ability of nanoplatforms to penetrate the brain in a non-invasive or minimally invasive manner [16,20]. The second advantage of using nanocarriers for brain drug delivery is the stability of the payload in the circulation prior to the delivery at the intended target site, thereby reducing the occurrences of undesired side-effects. Third, nanoplatforms (particularly the novel genre of smart nanoengineered carriers; see Section 4) offer avenues for spatiotemporal control of targeted drug delivery and action, especially in the case of external stimulus-activated versions of nanoplatforms [21,22,23]. Lastly, the unique inherent (as well as exogenously incorporated imaging agent-mediated) optical and/or electromagnetic features of some of the recently developed state-of-the-art nanocarriers offer effective diagnosis and monitoring of brain tumors.

Nevertheless, while NPs have been in clinical use for other cancer types, their applications in neuro-oncology have been somewhat delayed. Section 2 deals with the hindrances for nanodelivery of therapeutic and imaging agents to the brain. The state-of-the-art strategies for bypassing the BBB/BBTB, and the recent advances in bioengineering of nanocarriers to circumvent these hurdles, are discussed in Section 4 and Section 5, respectively. While this review does not include an exhaustive list of all the studies in nanotherapeutics for brain cancer research, the authors have provided relevant examples of the state-of-the-art innovative strategies for the design and employment of smart next-generation nanocarriers for brain cancer theranostics. In doing so, the hope is that this review can aid scientists in identifying the research gaps and act as an inspiration for the further design of innovative nanomedicines for the diagnosis, monitoring and treatment of neurological disorders in general, and brain cancers in particular.

## 2. Nanodelivery to the Brain: The Major Challenges

### 2.1. Blood Brain Barrier (BBB)

The blood brain barrier is the most important physiological component maintaining brain homeostasis. The BBB is a highly selective barrier lining the blood vessels in the CNS, acting as the first physical barrier against the potential toxic effects of both endogenous and exogenous chemicals, as well as in the regulation of the movement of essential nutrients and metabolites between the systemic circulatory system and the brain cells. Its protective role against the toxicity of molecules to the CNS and its formidable physico-chemical and electrostatic barriers in therapeutic medicine research are well-known. Indeed, BBB-mediated regulation of the transport of both endogenous and exogenous chemicals between the blood and the brain poses enormous challenges to neuro-therapeutic and diagnostic agents [24,25].

The BBB is comprised of highly specialized endothelial cells present in the brain capillaries, brain microvascular endothelial cells (BMECs) and other perivascular cells (pericytes, astrocytes and perivascular macrophages) that surround the BMECs. Each cellular component of the BBB has specialized functions [26,27]. While the BMECs lack fenestrations, limiting the diffusion of small molecules and proteins, the inter-endothelial tight junctions in BMECs regulate both transcellular (through the cells) and paracellular (between adjacent cells) movement of metabolites [28]. Efflux transporters such as P-glycoprotein on BMECs offer further hindrances to the retention of undesired substances in the CNS. The astrocytes at the basal lamina of the brain capillaries provide the cellular connections to neurons, while the pericytes regulate blood flow in the blood vessels. Lastly, perivascular macrophages elicit an immunomodulatory role.

Apart from small molecular compounds, such as water, and gaseous molecules, such as carbon dioxide, which passively diffuse across the endothelial cells of the BBB, other metabolites have to employ specialized transporters to gain access through the BBB [29,30]. Passive transport across the BBB occurs through tight junctions (water-soluble metabolites) or through the endothelium (lipophilic metabolites). Active transport, however, involves transporter- and receptor-mediated transcytosis and adsorption-mediated transcytosis [31]. The complexity and the highly selective permeability of the BBB pose significant hindrances to entry of therapeutic drugs into the CNS. However, utilizing one of the several transporter/receptor systems of the BBB, drug entry into CNS is potentially feasible. This has been one of the major achievements of nanoengineered carriers designed for optimized inherent BBB-penetrating capacity (Section 4.3). Some other smart nanomedicines have utilized external stimulus-activated brain penetration via transient and localized disruption of the BBB (Section 4.4).

### 2.2. Blood Brain Tumor Barrier (BBTB)

Several studies have indicated that neuropathologies can alter the structure, organization and function of the BBB [26,28,32]. Thus, in high grade brain malignancies, formation of the BBTB is often observed when cancer reaches a particular stage. The invasion of tumor cells into surrounding tissues disrupts the BBB, resulting in the BMEC-mediated formation of BBTB with the concomitant replacement of the BBB. The neovascularization of the BBTB is characterized by an abnormal and disorganized network of new blood vessels and brain tumor capillaries [33]. In such cases, the BBTB becomes the major obstacle for anti-cancer drug delivery. The high-energy requirement of BBTB formation is often involved with the increased expression of vascular endothelial growth factor (VEGF) for pathological and uncontrolled angiogenesis of blood vessels [34]. Intriguingly, these pathological features of the BBTB can be potentially exploited for drug delivery and therapy [35,36]. Thus, a humanized monoclonal antibody against VEGF has recently been approved by the FDA based upon its improved response rates and retardation of tumor progression, although these effects have been modest and temporary, and sometimes with severe adverse side-effects [37]. Nevertheless, nanotechnological exploitation of the pathological features of the BBTB, particularly VEGF-targeting for specific drug delivery at the tumor site has been a topic of several recent studies (Section 4.4).

### 2.3. Enhanced Permeability and Retention (EPR)

The BBTB is itself disrupted with the progression of the brain tumor leading to a leaky vasculature and resulting in the pathophysiological phenomenon of EPR, which is characterized by the formation of gaps in the BMECs [38]. This leakiness of the BBTB can be potentially utilized for delivery of NPs to the tumor site [39,40]. In particular, NPs can be engineered to take advantage of the EPR phenomenon for optimized theranostic approaches (simultaneous delivery of imaging and therapeutic agents in a single nanoplatform) [41]. However, it is important to note here that the utilities of drug delivery approaches utilizing the EPR effect is contested, with reports arguing against the passive targeting through the porous BBTB alone as an efficient strategy [42,43]. The inhomogeneous nature of the tumor vasculature and non-uniform disruption of the BBB/BBTB (porous BBTB within the tumor and an intact BBB at the periphery) furthers impedes the employment of EPR alone for drug targeting and delivery [44,45]. Further, using both computational modelling and imaging analyses, Sindhwani et al. [46] have provided evidence for active transcytotic mechanisms as being the predominant pathway of NP delivery across the disrupted BBB/BBTB in solid brain tumors, rather than the inter-BMEC gaps.

Of particular note, other endogenous mechanisms associated with cancer pathology may also be targeted for delivery of NPs to the tumor environment. For example, tumor-associated macrophages have been recently proposed as an alternate and effective strategy for drug delivery to cancerous cells [47]. However, more research studies need to be undertaken to confirm the effectiveness of such strategies for therapeutic delivery to brain-localized cancer cells.

### 2.4. Tumor-Specificity

Specific targeting of the drug to cancerous cells is a hurdle that is common to all types of cancers. The challenge for any systemic or localized treatment here is to limit the damage to noncancerous healthy tissue. In this regard, NPs have prominent advantages as they can be composed in a versatile manner with inclusion of biochemical moieties that enable specific recognition of cancerous cells (without affecting the normal ones) [45]. One or more targeting agents may be incorporated in the NPs, for example, to induce localization and then to induce the release of the drug payload at the intended tumor site. For example, hyaluronan, a ligand for cell surface glycoprotein CD44, which is heavily expressed on the surface of malignant brain cells, can be incorporated in the NPs for specific delivery to cancerous cells [48,49]. Readers are directed to a detailed review of peptides that can be employed for functionalization of NPs for their specific targeting of glioma cells [50].

## 3. Suitability of Smart NPs for Brain Cancer Treatment

As stated, when it comes to brain cancers, conventional therapeutic options offer modest therapeutic benefits, poor tendencies to delay progression/recurrence and enhanced side-effects. In this regard, nanotechnology is increasing being perceived as useful alternatives for both the imaging and therapy of brain cancers. Recent advances in nanotechnology and bioengineering have accelerated the use of NPs in the treatment of cancers and other brain pathologies, such as neurodegeneration [1,19,51,52]. A wide range of studies has provided evidence that smart nanoformulations with their versatile and multifactorial platforms may represent a particularly advantageous strategy for improving the delivery, safety and efficacy of therapeutic and diagnostic agents into the CNS [35,53]. Indeed, NPs harbor the unique and noteworthy capability of achieving combinatorial theranostic efficiency in a single formulation (for reviews, see [15,54]).

The superiority of nanomedicines for oncological applications relies on the ability to finely calibrate the physico-chemical properties of the NPs: size, shape, surface charge and brain tumor targeting [55]. In particular, NPs have the potential to harbor unique BBB-penetration capabilities, allowing better tumor targeting and reducing non-specificities [18,19]. Nanocarriers also enhance the solubility of the bioactive agents and protect them from in vivo degradation, increasing both their half-lives and bioavailability. Moreover, NPs can be optimized to improve drug release and pharmacokinetics, while simultaneously preventing non-specific effects [56,57]. Interestingly, physiological (pH, redox status, temperature) and non-physiological (ultrasound, electromagnetic field, light) variables can be utilized to facilitate the spatiotemporally regulated release of the drug payloads in response to the appropriate endogenous (e.g., pH and oxidative species) and exogenous (e.g., magnetic field and light) stimuli. Additionally, NPs hold great promise for efficient tumor accumulation, and hence precise tumor imaging and monitoring using payloads for fluorescent, photo-acoustic and Raman imaging [58,59].

One of the major theranostic advantages of nanomedicine in cancer therapy is that it is not limited to chemotherapy. Indeed, with the advent of newer tools and techniques and advancements in biological science research, some novel therapeutic strategies have arisen, including immunotherapy, gene therapy, oncolytic virotherapy and protein therapy (reviewed in [35]), which can be potentially used for brain cancer theranostics. Thus, in addition to the felicity of nanocarriers to carry varied bioactive chemotherapeutic agents, smart bioengineering approaches have the potential to broaden and optimize several novel treatment options.

Lastly, because of the versatility of the inherent physico-chemical properties as well as the unlimited prospects for manipulative bioengineering, nanomedicinal formulations have tremendous potential in drug-free therapeutic approaches [16]. In particular, novel phototherapeutic approaches [60], such as photodermal (therapeutic agents transform near infrared radiation into heat for ablation of hyperthermia-sensitive cancerous cells) and photodynamic (photosensitive agents generate oxidative species upon incidence of light leading to local cytotoxicity) as well as sonodynamic [61] therapeutic strategies for neuro-oncological applications, can benefit greatly from next-generation nanomaterials and nanocarriers. Advances in nanobiotechnology have also accelerated another novel line of therapeutic intervention, namely, the magnetic field-responsive or the magnetic hyperthermia therapy, which relies on the properties of magnetically functionalized NPs to convert magnetic energy into heat for the selective cytotoxicity of tumor cells [62,63,64,65]. While magnetic hyperthermia therapy offers greater tissue penetration, most of the current nanocarriers (such as commercially available Nanotherm® used for malignant gliomas) have to be injected intracranially for localization at the tumor site. Systemic delivery of magnetic nanocarriers for targeted brain cancer therapy could immensely benefit from their nano-optimization.

In conclusion, while it is clear that recent advances in smart bioengineering of nanoplatforms have provided huge promises for therapeutic, diagnostic and theranostic successes in clinical neuro-oncology, further research is warranted both for optimization of the strategies and for their comprehensive evaluation in clinical settings.

## 4. Contemporary Methods to Bypass the BBB/BBTB Barrier

**Figure 1:**
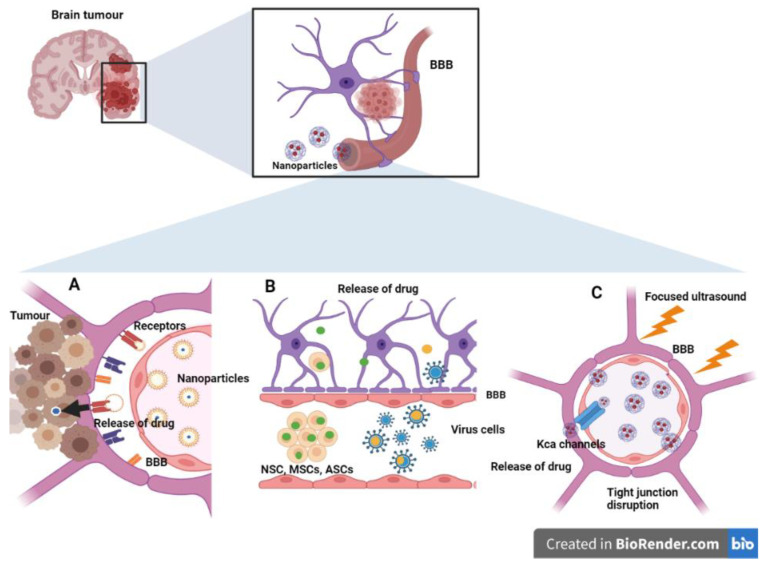
**Smart nanoengineering approaches to circumvent the blood brain barrier (BBB).** (**A**) Transcellular and paracellular transport pathways. Increased expression of receptors such as transferrin, integrins and LDL receptors facilitate the receptor-mediated uptake of drug-carrying nanoformulations. (**B**) Cell- and viral-mediated BBB crossing. Various stem cells, neutrophils, and viral vectors can cross the BBB carrying nanomedicine. (**C**) Physico-chemical (transient) disruption of the BBB. Focused ultrasound can decrease the structural proteins of tight junctions, namely, claudins and occludin. It can also increase the expression of calcium-activated channels for the delivery of nanomedicine. (ASCs: Adipose-derived mesenchymal stem cells; BBB: Blood brain barrier; BM-MSCs: Bone marrow-derived mesenchymal stem cells; LDL: Low-density lipoprotein; MSCs: Mesenchyma stem cells; NSCs: Neural stem cells; KCa: Calcium-activated potassium channels).

As discussed, tumor targeting to CNS sites bypassing the BBB/BBTB barriers at the required therapeutic concentrations, while reducing non-specific effects on the peripheral and non-tumor cells, is the major challenge in neuro-oncology. There are several potential techniques for bypassing the BBB for delivery of NPs at the tumor site in the CNS (Figure 1).

### 4.1. Intracranial Local Delivery

The intracranial pathway involves the direct and localized delivery of the drug/imaging agent to the tumor site (reviewed in [66,67]). Several approaches to intracranial drug delivery are known: post-surgery intracerebral implantation, intracerebroventricular infusion and convection-enhanced diffusion (CED). In particular, the intracerebral implantation of nanopolymers that regulate the localized release of active drugs at high concentrations at the tumor site is well known [68]. Interestingly, this also helps in the protection of the drug from degradation and from the clearance pathways of the immune system [69,70]. NPs delivered by CED on the other hand seem to result in a heterogeneous distribution of brain penetrating NPs, resulting in their substandard tumor specificity and accumulation [71], although incorporation of NPs with tumor targeting agents such as chlorotoxin may circumvent this particular problem [72]. However, intracranial routes for localized and directed delivery to brain tumors in general may not be as efficient as previously thought. Indeed, the BBB/BBTB is not the only hurdle that needs to be bypassed; other factors such as interstitial fluid flow and efflux mechanisms at the BBB/BBTB also need to be addressed [73,74]. Here, combining nanomedicines with the local intracranial delivery strategies may be promising; however, one should be aware that invasive procedures and the equipment/skill involved may overweigh the therapeutic benefits, particularly with regard to some of the recently developed non-invasion and less-invasive delivery routes (discussed in Section 4.3 and Section 4.4).

### 4.2. Intrathecal Delivery

Infusion of drugs in the intrathecal space (filled with cerebrospinal fluid; CSF) either in the Ommaya reservoir or directly into the CSF in the spinal column has been a known alternative for bypassing the BBB [75]. While the clinical relevance of this route in cancer and non-cancer pain therapy is known [76,77], its applications have been rather limited in the therapy of CNS tumors. This is because of the invasiveness of the technique and the passive nature of the drug delivery, which relies on normal CSF flow (which is often not the case in brain metastases). For obvious reasons, however, the intrathecal route of delivery of drug/imaging agents may have greater implications for leptomeningeal and spinal metastases [78].

### 4.3. Intranasal Delivery

The intranasal mode of drug delivery is made possible by the unique and direct nose-to-brain route through the olfactory area of the nasal sub-mucosa to the CSF, thus bypassing the BBB [79]. Brain delivery of intranasally administered agents may also rely on the olfactory and trigeminal neural pathways between the nasal mucosa and the brain [80]. Intranasal administration of targeted therapeutics to brain tumors is a fast and non-invasive method of drug delivery with reduced systemic exposure and side effects [81,82]. Not surprisingly then, the intransal administration of NPs for the treatment of brain tumors has been proposed and may constitute an attractive non-invasive option of drug delivery to the CNS tumors [83,84,85]. For instance, NPs functionalized with the anti-ephrin type-A receptor 3 (EphA3) antibody were found to be effective in glioblastoma targeting when delivered intranasally [86,87]. The effectiveness of an intranasally delivered perillyl alcohol-based medicinal strategy against brain tumors [88,89] has generated tremendous interest and has propelled research interest in the application of intranasal routes for drug delivery to the CNS for neuropathologies in general [90] and CNS cancers in particular [91,92].

It should be noted, however, that this technique of delivery is still relatively nascent, and as such more studies are warrantied for further evaluation of its suitability for neuro-oncological purposes. For example, the intranasal route of drug delivery may be limited by the dosing volume through the nasal cavity and spillage (and hence the amount of drug entering the CNS). Moreover, the accumulation and curative activity of drugs delivered through the intranasal route are critically dependent on the location of the tumor tissue in the CNS. In this regard, several aspects of intranasal delivery can be optimized by nanobiotechnological strategies, including minimizing clearance/degradation by mucosal enzymes, increasing target-specificity (e.g., by ligand incorporation), and finer control of the spatiotemporal aspects of release, for example, by endogenous (pH and reactive oxygen species) or external (magnetic field and ultrasound) stimuli.

### 4.4. Novel Systemic Delivery Approaches

Since the brain is one of the most highly perfused organs with a dense vascular network, minimally invasive systemic delivery of therapeutics can be a realistic option if appropriate strategies are employed to bypass the BBB/BBTB barriers and to reduce non-specific peripheral distribution. Because of their versatility and manipulative potential, NPs are tremendous prospective candidates in this respect. In fact, the systemic delivery of NPs remains the most researched route for brain cancer therapies, and several possible strategies have been proposed to increase their efficiency for brain tumor targeting.

#### 4.4.1. Transcellular and Paracellular Transport Pathways

Systemic intravenous administration of NP functionalization with suitable targeting ligands can aid in their BBB/BBTB penetration, exploiting both the transcellular and the paracellular pathways [35,93]. A logical approach is to harness the ability of the endogenous receptors on BMECs of the BBB for receptor/carrier-mediated transcytosis of NPs. Several studies have proposed receptor-mediated transcytosis as a feasible pathway of drug entry across the BBB [94,95]. For example, drugs incorporated with mannose have been shown to cross the BBB using the glucose transporter (GLUT) [96]. Other receptors can also be efficiently employed for this purpose, including transferrin receptor, low-density lipoprotein (LDL) receptor and insulin receptor (IR) [97]. In particular, because of its high expression on endothelial cells of the BBB, transferrin receptor has served as an excellent target for CNS entry of drugs conjugated with the transferrin or anti-transferrin monoclonal antibody [98,99]. It is interestingly to note here that in vitro transcribed mRNA (IVT-mRNA) systems can be complemented with nanoengineered particles for the endogenous expression of antibodies, thereby bypassing the need for large scale production, purification and conjugation of exogenously prepared antibodies [100].

In addition to the transferrin receptor ligands/antibodies, ligands against LDL receptors on endothelial membranes can also be harnessed to facilitate the CNS uptake of bioengineered functionalized NPs. Indeed, examples of the ligands for LDL receptors coated on NPs abound in the literature (e.g., Angiopep [101] and apolipoprotein E [102]; for a detailed review, see [103]). Similarly, IR ligands have also been employed for stimulating the entry of NPs in the CNS [104]. Intriguingly, the relatively low endocytotic rate of ligand receptors on the BMECs (compared to peripheral endothelial cells), which incidentally is a prominent feature of the high impermeability of BBB, can be harnessed for specific nanodelivery of therapeutics to the brain. Indeed, Gonzalez-Carter et al. [105] used this strategy for brain-specific localization of biotinylated ligands via avidin-functionalized nanomicelles with minimal non-specific peripheral accumulation.

Integrins, extracellular matrix (ECM) transmembrane receptors associated with tumor progression, invasion and neovasculation, constitute an interesting target of receptor-mediated transcytosis utilizing the pathological EPR effect. In particular, ligand-targeting strategies for α_V_β_3_ and α_V_β_5_ types of integrins have been employed in nanomedicine research for neuro-oncology applications [106]. It is interesting to note here the curious case of the tripeptide sequence of arginine–glycine–aspartate (RGD), which is found in many ECM proteins for the recognition of several of the integrins (including the α_V_β_3_ and α_V_β_5_ types). Accordingly, NPs functionalized with the cyclic RGD peptides offer promising alternative targeting strategies for therapeutic agents in glioma [35,107].

Lastly, examples of adsorptive transcytosis-mediated drug entry utilizing conjugation with cationic proteins or cell-penetrating peptides are also known in the literature [108]. The adsorptive uptake of lipid-based NPs coated with surfactants by the BBB has also been experimentally known for some time [109,110,111]. While these NPs containing the BBB-penetrating agents do not lead to physical disruption of the BBB, their utility in drug delivery to brain cancers is limited by their non-specific nature and by the size of the NPs. On the other hand, paracellular transport of nanomedicines relies entirely on the malignancy-related loss of BBB/BBTB integrity and the ensuing EPR effect. The transport through this pathway and its pitfalls are discussed in Section 2.3.

#### 4.4.2. Physico-Chemical (Transient) Disruption of BBB/BBTB

To address the non-uniformity of the tumor vasculature (Section 2) and hence the inadequate NP accumulation at the intended site and poor therapeutic benefits, research has been directed to strategies that can disrupt BBB/BBTB integrity in a localized and transient manner (reviewed in detail in [112]). Strategies can be employed for the disruption of the BBB, for example, by increasing local osmotic pressure by hyperosmol mannitol infusion [113]; however, this method is generally inconsistent and non-specific for tumor BBB/BBTB [114].

An interesting smart strategy is the use of focused ultrasound (FUS) for temporary disruption of the BBB and hence convenient delivery of NPs to gliomas [115,116]. This state-of-the-art so-called microbubble-enhanced diagnostic ultrasound (MEUS) technique is based upon the transient and reversible structural changes in the BBB induced by the decrease in the major structural proteins of the tight junctions, claudins, occludin and junctional adhesion molecules upon ultrasound irradiation and microbubbles [117]. In addition, the increased expression of calcium-activated potassium (K_Ca_) channels [118] and the reduced expression of p-glycoprotein [119] have also been recently implicated as additional pathways of BBB disruption in MEUS. The detailed review of the basic principles and potential application of MEUS can be visited in excellent review articles [120,121,122]. While this technique is still in its infancy in the neuro-oncology field, there have been some notable examples of positive outcomes in the literature. For example, doxorubicin, a chemotherapy agent, was successfully delivered to tumor sites in the brain via MEUS [123]. Interestingly, a recent study employed the MEUS strategy for transient disruption of the BBB and the targeted delivery of clustered regularly interspaced short palindromic repeats-associated protein 9 (CRISPR/Cas9) plasmids targeting the drug-resistance gene, O6-methylguanine-DNA methyltransferase (MGMT) in a glioblastoma model. The authors were very successful in targeting the therapeutic NPs to an ectopic tumor in mice and in decreasing the MGMT-mediated resistance to the chemotherapeutic agent temozolomide [124]. In fact, smart bioengineering approaches based upon the CRISPR/Cas9 system have the potential to become effective tools for the targeted delivery of genome-modifying agents to the CNS for alteration of the gene expression profiles of cancerous cells [125].

Another novel technique for transient BBB/BBTB disruption and facilitation of drug delivery in the brain is the transcranial magnetic stimulation and consequent NMDAR-mediated neuronal activation [126]; however its application in neuro-oncology has been limited so far [127]. Similar use of chemical enhancers of BBB permeability has also been proposed for CNS drug delivery [128,129]. Unfortunately, this strategy has so far not yielded successful results in clinical studies [130,131].

#### 4.4.3. Cell- and Viral-Mediated BBB Crossing

The ability of some cell-types to migrate towards malignant tumor cells in the brain without attracting immunological responses can provide an interesting option for drug delivery by NPs. The various cells that can be employed for this purpose include neural stem cells (NSCs) [132], bone marrow-mesenchymal stem cells (BM-MSCs) [133] and adipose-derived mesenchymal stem cells (ASCs) [134]. Loading these cells with therapeutic nanomedicines can be a successful strategy for optimal targeting to the tumor cells in the brain [135,136]. Similarly, neutrophils (peripheral immune cells) can also be engineered for optimized drug delivery to the brain tumor [137]. For a detailed visitation of the approaches, applicability and limitations of neural stem cell-based therapies in neuro-oncology, refer to a recent review by Benmelouka et al. [138]. Drug delivery through viral vectors, based upon their known efficiency in brain targeting, has also shown some potential in mitigating the impermeability of the BBB. While there are numerous examples of viral vectors and virally derived ligands used for brain cancer therapies (for detailed reviews, see [139,140]), their clinical applications may be limited.

## 5. Overview of the State-of-the-Art NPs for Neuro-Oncology Applications

**Figure 2:**
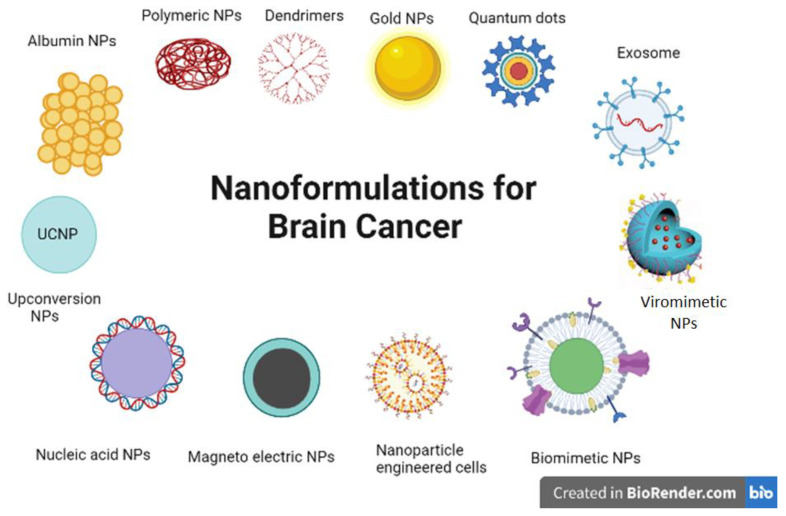
**Novel nanoplatforms currently under research for neuro-oncology applications.** The figure illustrates some of the major categories of nanoplatforms that are currently being researched for improved and effective diagnosis and therapy of brain cancers.

It is clear that there has been an exponential increase in the development of potentially successful strategies to allow penetration through the BBB/BBTB, raising hopes for effective brain drug delivery pathways in clinical settings. In this regard, nanocarriers have emerged as the most suitable candidates for mediating the delivery of diagnostic, therapeutic, theranostic agents (Figure 2). Regrettably, clinical applications of brain-directed nanomedicine is still lagging behind, and not even a single CNS nanoformulation has been approved for clinical neuro-oncological purposes [141]. Nonetheless, recent advances in smart bioengineering strategies has accelerated the surge for designing and evaluating non-invasive therapies of neurological disorders, including brain tumors [142], which might ultimately lead to the successful development of one or more theranostic strategies in the future.

In recent decades, several kinds of NPs have been studied in in vivo and in vitro models of cancers. For example, the veteran liposomal nanoformulations have been widely used in non-CNS clinical practice since their inception in 1995. These nanoformulations have excellent credibility as safe and versatile agents for the encapsulation of various drugs (both lipophilic and hydrophilic), and of a wide range of molecular weights. Moreover, the drugs are not required to be chemically modified for encapsulation [36,143]. Use of liposomes in brain cancer therapy has also been proposed; for instance, functionalized liposomes with targeting ligands, such as those against the transferrin receptors of the BBB, have shown promising results in brain tumor therapy [144,145]. For this particular review, however, we only focus on the recently developed and next-generation multi-functional nanomaterial formulations that have garnered prominent attention for potential clinically relevant diagnostic and therapeutic applications in brain cancer theranostics. It should be noted that while this section is divided into different subsections for ease of comprehension, this classification is not exclusive, and several examples of engineered hybrid/combinatorial nanocarriers (simultaneously falling in different categories of NP types) in neuro-oncology research are discussed throughout.

### 5.1. Polymeric NPs and Dendrimers

Polymeric NPs are a class of nanocarriers composed of natural or synthetic polymers. Their main advantage lies in their inherent biocompatible, non-immunogenic and biodegradable characteristics and the ability to entrap hydrophobic drugs in the hydrophobic core, protecting them against endogenous degradative and clearance mechanisms. Though they have been long thought to harbor the potential for therapy of brain cancers, significant challenges must be identified and addressed (reviewed in [146,147]).

In the neuro-oncological perspective, carbohydrate-based polymeric NPs have been proposed as platforms with significantly improved brain delivery agents. In addition to the ease and cost-efficiency of their preparation and their inherently biodegradable and biocompatible nature, the ability to functionalize carbohydrate polymeric NPs with diverse groups of ligands offers multiple pathways of therapies and imaging strategies for brain cancers [148]. In particular, the natural polysaccharide chitosan (and its derivatives) has been a focus of the nanotechnological approach in the therapy of brain cancers, as it offers protection from endogenous degradative mechanisms and elicits properties of controlled release and enhanced bioavailability, while allowing efficient penetration across the BBB/BBTB [149,150]. Moreover, surface modification of chitosan-based NPs, e.g., by functionalization with transferrin, apoE or chlorotoxin, can improve brain tumor targeting [151,152]. Hybrids of chitosan/supramagnetic NPs have also been shown to elicit increased brain tumor targeting and cytotoxicity [153]. Interestingly, Khan et al. have developed a nanolipid chitosan hydrogel for non-invasive nose-to-brain drug delivery [154]. A nanoengineered hybrid Au/chitosan formulation has also shown promising results in photothermal therapy of glioblastoma [155]. Hence, it is clear that novel bioengineering of chitosan-based NPs can offer diverse (and combinatorial) approaches in the imaging and treatment of brain cancers (reviewed in detail in [156]).

Dendrimers (also called dendritic NPs) are a novel class of synthetic polymeric smart nanoformulations that have recently garnered immense interest for neuro-oncology applications. These highly branched polymers with modifiable surface functionalities and available internal cavities with a high loading capacity are emerging candidates as nanocarriers with immense potential for a directed theranostic approach to brain cancer imaging and therapy [157,158]. Owing to their unique heavily branched nature and increased surface area, dendrimers possess the distinctive ability to incorporate multiple surface functionalizations. However, research evaluating their therapeutic and diagnostic potential in neuro-oncology has been limited so far, with only a few studies evaluating their efficiency in brain cancer therapy in vivo. For instance, a polyamidoamine dendrimer–chitosan conjugate was employed for delivery of a chemotherapeutic agent with the observation of improved glioma cytotoxicity in vivo [159]. Similar observations of increased in vivo anticancer activity of polypropyleneimine dendrimer loading with another chemotherapeutic agent have also been reported [160,161]. Of note, hyperbranched polyermic NPs, which are slightly different from dendrimers in topological structures, have also been recently proposed as delivery agents for chemotherapeutic agents at brain cancer sites [162].

### 5.2. Albumin NPs

Similar to natural carbohydrates, protein-based NPs offer a convenient-to-formulate, low-cost, biocompatible and biodegradable pathway of drug delivery to the brain. Moreover, they harbor immense potential for functionalization by a wide range of ligands and therapeutic/imaging agents for a targeted approach in brain cancer treatment. An appropriate example is the transferrin-based NPs that have elicited tremendous abilities to cross the BBB/BBTB by targeting the transferrin receptor-mediated transcytosis (see also Section 4.4.1) for brain tumor therapy [163,164]. Casein (and its peptides) is a natural food ingredient that can be employed in nanoplatforms for brain cancer therapeutics because of the former’s brain-targeting capability [165]. This has indeed recently been shown to be the case in vivo in glioma-bearing mice [166]. Interestingly, the ability of casein and its peptides to penetrate the BBB may be exploited to engineer milk exosome-based nanodevices (see Section 5.10), utilizing an oral route for brain targeting, particularly when considering the biocompatibility and resistance of milk exosomes to the harsh acidic and degradative conditions in the gut [167,168,169].

One of the most widely used protein-based nanoformulations in the therapeutic studies of brain cancers involves the use of serum albumin, possibly due to the high energy/nutrient requirement-induced overexpression of albumin binding proteins (e.g., gp60) on cancerous cells [170,171]. Cationized albumin, in particular, has been shown to infer greater ability of the NPs for uptake by BBB endothelial cells with little toxicity [172]. Albumin NPs are particularly beneficial for increasing the circulation time and hence the bio-availability of the encapsulated drug. Several examples of smart bioengineered albumin-based nanocarriers are available in the literature. Thus, albumin NPs functionalized with natural brain-penetrating enhancers obtained from traditional Chinese medicinal products were found to effectively pass the BBB and accumulate in glioma tissue with negligible peripheral cytotoxicity [173]. Nanoengineered hybrid ferric oxide NPs coated with albumin and functionalized with monoclonal antibodies against VEGF were shown to be both stable and capable of efficient MRI-assisted imaging of intracranial gliomas [174]. In fact, the formulation of hybrid magnetic NPs employing gadolinium oxide, supramagnetic iron oxide or manganese oxide; and bovine serum albumin is thought to be a very promising minimally cytotoxic strategy for theranostic targeting and MRI imaging of brain gliomas [173,174,175].

### 5.3. Gold (Au) NPs

Gold-based nanoconjugates synthesized on Au cores come in different shapes and have some unique properties that can be harnessed for brain cancer treatment. In addition to their optimal size, Au NPs are biocompatible and can be conjugated with appropriate cell-targeting ligands [176,177] and can potentially be nanoengineered for time- and dose-optimized drug release [178,179,180]. Applications of smart Au-based hybrid NPs in neuro-oncology research abound in the recently published literature. Thus, an anti-transferrin receptor antibody [181,182] and glucose [183] have been employed for targeted delivery of Au NPs in the mouse brain. The suitability of epidermal growth factor- [184] and chlorotoxin peptide-functionalized [185] Au NPs for increased tumor retention in the rodent brain has also been evidenced. Intranasally delivered immunotherapeutic oligonucleotide-functionalized [186] as well as anti-EphA3 antibody-functionalized [87] Au NPs have been successfully employed for glioma accumulation and therapy. Interestingly, Au NPs have also been employed for targeted miRNA-mediated gene therapy of glioma cells in vivo [187]. Hybrid polyethyleneimine-entrapped Au NPs functionalized with RGD peptide have been used for siRNA-mediated gene-therapy-mediated cytotoxicity of glioblastoma cells [188]. A similar nanoformulation of fluorescein isothiocyanate labeled polyethyleneimine/Au NPs has been proposed to elicit excellent potential for simultaneous imaging and transport of chemotherapeutics against glioma cancer cells [189]. Hybrid nanoshells with a dielectric core composed of silica and coated with Au were loaded with macrophages for the efficient infiltration of glioma spheroids; moreover, the Au coating absorbed near infrared light and converted it to heat for photothermal ablation of the cancer cells [190]. In conclusion, it is quite clear that Au-based NPs, particularly those engineered for optimized biostability, targeting and delivery offer a wide range of therapeutic and imaging options in neuro-oncology research.

### 5.4. Magneto-Electric NPs (MENPs) and Magnetosomes

The magnetic and electric properties of the MENPs (such as those employing gadolinium and iron oxide cores) aid in specific targeting to the tumor site and can also be utilized for magnetic resonance imaging (MRI) traceability [63]. Further, the ease and cost of generation of MENPs along with their biocompatible characteristic make them popular candidates for neuro-oncology applications. Improved brain penetration of MENPs by smart bioengineering approaches has been shown in a number of animal studies, for instance, using functionalization with cell-penetrating peptide Tat [191] and using electromagnetic field stimulation [192,193]. Interestingly, MENPs composed of a CoFe_2_O_3_ core were found to be effectively delivered to the brain of a non-human primate with little toxicity [194]. In addition to the application of the external magnetic field for delivery of the magnetic NPs across the BBB, combining other physical stimulus strategies such as focused ultrasound can further improve the targeted delivery of MENPs [195,196].

In particular, the unique ability of MENPs for localized hyperthermia therapy harbors huge implications for brain cancer theranostics (reviewed in [62,64]). In fact, recent studies provide evidence for the huge potential of iron oxide NPs for theranostic delivery in neuro-oncology [197]. For instance, Fe_3_O_4_–Au composite magnetic NPs were used for a combinatorial magneto-photothermal (magnetic fluid hyperthermia and near infrared hyperthermia) therapeutic approach against glioma cells [198]. Nanoengineered hybrid chitosan–dextran–supramagnetic iron oxide NPs were shown to elicit simultaneous MRI imaging and cytotoxicity of orthotopic glioma cells in rats [153]. Aminosilane-coated superparamagnetic iron oxide NPs were shown to have therapeutic potential in gliomas in vivo [65,199].

Another interesting class of smart magnetic NPs that could potentially prove to be beneficial in neuro-oncology research are what are known as the magnetosomes, which are membranous structures derived from magnetotactic bacteria containing iron-rich magnetic NPs. They are a particularly relevant alternative to traditionally synthesized iron oxide magnetic NPs with regards to their increased biocompatibility while maintaining the hyperthermia and tracing properties [200,201]. Moreover, magnetosomes can be surface-functionalized with different peptides and proteins and also engineered for the controlled release of endotoxins that attract immune cells. Thus, the superior therapeutic effects of nano-engineered magnetosomes against glioma cells in vivo were observed both because of enhanced photo hyperthermia and the controlled release of endotoxins [202]. In addition, enhanced magnetic hyperthermia mediated by magnetosomes when induced by an alternating magnetic field has also been proposed as an alternative therapeutic strategy for brain cancers [203,204]. Similarly, RGD-functionalized magnetosomes have been shown to confer greater susceptibility of brain tumors for radiotherapy by X-rays and protons [205].

### 5.5. Quantum Dots (QDs) and Carbon Quantum Dots (CQDs)

Quantum dots are luminescent/fluorescent semi-conducting nanovectors having unique optical properties and immense potential for surface chemistry alterations, making them exceptional candidates for traceable and targeted delivery of diagnostic, therapeutic and theranostic agents [206,207,208]. The aqueous stability and biocompatibility of QDs can be a limitation; however, this can potentially be overcome by functionalization with different materials [209,210]. Smart bioengineering of QDs can increase their efficiency of BBB penetration and brain targeting, as shown by many experimenters [211,212,213,214,215,216,217]. Bio-conjugation of asparagine–glycine–arginine (NGR) peptides [212] or aptamer 32(A32) [218] on QDs was shown to allow efficient targeted fluorescence imaging of brain malignancy and tumor vasculature in vivo. The therapeutic potential of smart nanoengineered QDs and CQDs in in vitro glioblastoma cells has also been studied, for example, using the electrostatic conjugation of ZnS QDs with the chemotherapeutic agent doxorubicin [219] and transferrin-conjugated carbon dot incorporated with anti-cancer drugs [220,221]. Interestingly, CQD-based NPs functionalized with transferrin and loaded with chemotherapeutic drug doxorubicin elicited excellent cytotoxic effects against pediatric brain tumor cells in vitro [164]. Recently, fluorescent nanoformulations composed of a semiconductor core conjugated with a mitochondria-targeting peptide were employed for both imaging and inducing the death of brain cancer cells [222]. As a special mention, graphene QDs have immense potential in neuro-oncology owing to their higher biocompatibility, low toxicity and their inherent ability to traverse the BBB (reviewed in [223,224,225]). However, their applications in neuro-oncology have been so far limited to in vitro cell culture models [226,227].

### 5.6. Upconversion NPs (UCNPs)

Lanthanide upconversion nanoparticles are an interesting class of nanocarriers that convert deep tissue penetrating near-infrared light into visible/ultraviolet emissions [228]. As such, like QDs, they are excellent non-invasive candidates for combinatorial sensing and therapeutic nanomedicine [229]. UCNPs offer a wide range of relevant features for brain cancer diagnosis and treatment, including high biocompatibility and stability, sharp emission bandwidths, low background and high photostability and excellent potential for modulation of physico-chemical characteristics [230,231]. The convenience of shape and surface modification of UCNPs is particularly suited for BBB penetration. Hence, a hybrid UCNP-based organic nanoformulation loaded with photothermal sensitizers elicited pronounced uptake and cytotoxicity by astrocytoma cells in vitro [232]. Transferrin-conjugated UCNP-loaded liposomes have recently been shown to elicit efficient drug release and cytotoxicity in C6 glioma cells [233]. It should however be noted that the in vivo animal studies involving UCNPs have mainly focused on imaging and monitoring of brain tumors [234,235]. However, with further development of smart nanoengineered versions, one can expect an increase in the applications of UCNPs for multiple therapeutic and theranostic strategies in neuro-oncology research. Thus, in an interesting recent study, Teh et al. used an UCNP implant comprised of a poly(ethylene glycol) diacrylate core for wireless photodynamic therapy in a mouse xenograft glioblastoma model [236].

### 5.7. Nanoparticle-Engineered Cells and Biomimetic Strategies

Developments in the understanding of (and the techniques related to) stem cell biology have resulted in an exponential surge in research studies aimed at exploiting their biomedical applications [237]. In particular, their oncological applications as carriers for therapeutic agents have been followed rigorously because of their inherent ability of tropism and migration towards the tumor sites and the avoidance of the endogenous immunological pathways [238,239,240]. Novel strategies of nanoengineering of human stem cells may circumvent some of the common issues with conventional NPs, such as rapid clearance and limited circulation time for an efficient BBB penetration. Several cell types have been tested as a platform for bioengineering of NPs, including human hepatocarcinoma cells (HepG2), human cervical cancer cells (HeLa, SiHa), neural stem cells (NT2) and human embryonic kidney stem cells (HEK-293). In particular, mesenchymal stem cells (MSCs) are particularly suited for therapeutic delivery of bioactive agents because of their abundant availability and ease of extraction, feasibility of autologous transplantation and limited ethical concerns [241,242,243]. Readers are directed to detailed review articles delineating the advantages, feasibility and study examples as well as limitations and ethical considerations of using engineered stem cells for brain cancer therapy [241,244].

In recent years, the combination of nanotechnology and stem cell therapy has been put forward as an interesting option for brain cancer therapy with tremendous potential benefits. Nano-engineering of stem cells allows for optimization of the cellular microenvironment, brain penetration and in vivo survival [245,246]. For example, human adipose-derived mesenchymal stem cells engineered with biodegradable polymeric NPs and functionalized with bone morphogenetic protein 4 (BMP 4) was found to specifically target brain tumor-initiating cells [247]. Similarly, stem cells bioengineered to overexpress tumor necrosis factor-related apoptosis-inducing ligand (TRAIL) were employed to target and eradicate glioblastoma cells in an experimental animal model [248]. Interestingly, a magnetically powered stem cell-based smart microbot was recently shown to successfully penetrate the mouse brain using the intranasal pathway [249], opening up yet another potential non-invasive strategy for targeting and treating brain tumors. Cellular vaccination therapy is another smart biomimetic strategy that utilizes bioengineered T-cells, dendritic cell-based multipeptides and natural killer cells that can be applied for brain cancer treatment [250]; however, a thorough understanding of its therapeutic benefits and advantages in neuro-oncological applications need to be addressed first.

### 5.8. Viromimetic NPs

The unique and versatile morphological, biological and surface properties of viruses combined with their efficiency in escaping endogenous immune clearances and targeting of specific cells can potentially serve as a platform for the design of suitable NPs [251,252]. Not surprisingly, evaluation of the applications of viromimetic NPs in neuro-oncology is also gaining research interest. For instance, the extraordinary ability of rabies virus to infiltrate the CNS has been the basis of design of viromimetic NPs for both chemo- [253] and photothermal [254] therapies in in vivo glioma models. The human immunodeficiency virus-derived TAT peptide has also served as a viromimetic strategy for the delivery of NPs across the BBB for glioma therapy in several studies [255,256,257]. Interestingly, plant viruses such as cowpea mosaic virus [258] and tomato bushy stunt virus [259] can also serve as templates for the design of smart NPs with enhanced cytotoxicity in brain cancer cells; however, the BBB penetration ability of these NPs remains to be tested in vivo. For a detailed review of viromimetic NPs and their therapeutic potential in neuro-oncology research, refer to the review by Root et al. [53].

### 5.9. Nucleic Acid-NPs

With the advances in the basic knowledge of the molecular mechanisms of brain tumor pathology, several genetic targets have emerged, which in turn has increased the prospects of developing suitable gene therapy strategies in clinical settings of neuro-oncology. Conventionally, DNA delivery has relied on viral vectors; however, smart nanoplatforms offer several potential advantages, such as enhanced brain penetration, biocompatibility and flexibility of cargo capacity. Indeed, smart nanoengineering can potentially allow the delivery of DNA/RNA for a multi-targeted gene therapeutic approach against brain cancer. Various aspects of cancer cell biology can be targeted simultaneously; induction of the expression of tumor suppressor genes, reduction of the expression of oncogenes, stimulation of cell death programming, sensitization of the tumor cells for subsequent cytotoxic treatment, induction of differentiation of tumor-initiating cells and regulation of the proliferation and migration of cancer cells [260]. Interestingly, nucleic acid NPs can also be employed for immunotherapeutic (which involves modulation/stimulation of endogenous immune mechanisms against cancer cells) approaches in neuro-oncology [261,262]. Novel bioengineering approaches employing the CRISPR/Cas9 system (Section 4.4.2) may represent a potentially useful and effective strategy against CNS cancers [125].

Broadly, nucleic acid-NPs can be divided into DNA- and RNA-NPs. Ideally, the DNA-based NPs are expected to drive expression of genes that can induce cell death or make the cancer cells susceptible to other cytotoxic agents. RNA-NPs are more versatile in the payloads and can incorporate almost all of the known agents of RNA-based therapeutics: mRNAs, miRNAs, siRNAs and ribozymes. While miRNAs and siRNAs are gene therapeutic candidates for controlling the expression of particular endogenous mRNAs, mRNA-based NPs are increasingly been perceived as a tool for vaccine-based immunotherapeutic strategies against brain cancer [263,264]. For the detailed understanding of contemporary nanoengineering techniques of nucleic-acid-NPs and their applications in neuro-oncology, readers are directed to recent excellent review articles [262,265,266].

### 5.10. Exosomes

Exosomes are extracellular vesicles secreted by endomembrane-containing cells (most mammalian cells) and are implicated in intercellular communication, both under normal and pathological conditions [267,268]. The application of exosomes as delivery vehicles for therapeutic agents is similar to synthetic nanocarriers with the added benefit of their natural biological occurrence, biocompatibility, metabolic stability and non-immunogenic nature [269]. In the perspective of brain physiology, exosomes derived from brain ECs regulate exchange of molecules across the BBB for maintenance of brain functions [270]. Not surprisingly, the exosome-mediated delivery of therapeutic agents (such as small molecules, proteins and nucleic acids) across the BBB is known (see review [271]); however, their therapeutic applications in treatment/amelioration of neuropathologies, including brain cancers, has been rather limited. Yang et al. successfully delivered siRNA against VEGF using exosomes isolated from brain EC culture media across the BBB, resulting in the inhibition of VEGF in zebrafish brain tumors [272,273]. Recently, nanoengineered doxorubicin-loaded exosomes were found to be successful against glioma in vivo [274]. In this regard, exosomes derived from human and animal milk may serve as unique agents for oral delivery of therapeutics to the brain, with tremendous possibilities for therapy of various neuropathologies.

In conclusion, while it is clear that exosomes could potentially be a very important tool for the delivery of various kinds of therapeutic agents to the CNS across the BBB, more research studies are needed for a thorough understanding of the choice of exosome donor cell, the optimization of encapsulation procedures, the evaluation of loading efficiencies and toxicity and pharmacokinetic properties. It is relevant to stress the interesting potential utility of endogenous exosomes, particularly isolated from body fluids such as blood and CSF, in serving as targets for biomarker identification for neuropathological conditions, including brain tumors [275].

## 6. Conclusions

This review revisits some of the challenges in translational neuro-oncology research and discusses the recently developed smart nanotechnological strategies with the potential to overcome them. In spite of the tremendous potential of nanomedicine for the treatment of brain cancers as evidenced by preclinical animal experiments, the clinical human trial data has remained less than satisfactory. The reasons for this discrepancy are not quite understood, but it is speculated that the less-than-pronounced EPR effect and the heterogeneity of human brain tumors are the major contributors. There is clearly still hope and much room for improvement and optimization. For example, the variable ERP effect in humans can be circumvented by nanomedicines with better pharmacokinetics and tumor deposition. Combinatorial approaches (simultaneous therapeutic and imaging; and multiple therapeutic agents) and personalized medicine (theranostics) are the future lines of research that may prove beneficial for addressing the tumor heterogeneity issue (and drug resistance) in human brain cancers. In particular, the incorporation of imaging and therapeutic agents into single nanoplatforms may lead to significant clinical successes in dealing with brain cancers in a clinical setting. Stimulus-responsive strategies, especially focused ultrasound and magnetic field activation, may elicit the highest clinical potential, as they offer no tissue- or BBB-penetrating limitations. These and other smart bioengineered options for superior diagnosis, monitoring and treatment of brain cancers are already being rigorously perused by researchers globally (Table 1). More studies, however, are needed to evaluate (a) the effectiveness of the combined approach (in comparison to separate studies of the therapeutic and imaging agents, which has been the case for most experimental models); (b) the optimization of brain tumor targeting across the heterogeneous cerebral vasculature; and (c) the non-specific toxicity and safety of nanomedicine in humans. We can only hope that the next-generation nano-theranostics will efficiently fulfil the criteria of reduced toxicity, enhanced efficacy and sustained treatment for brain cancers, bringing us to the doorstep of clinical success in neuro-oncology.

## Figures and Tables

**Table 1:** **Smart nanomaterial formulations used in brain cancer theranostics**.

Type of Nanoparticles (NPs)	Characteristics	Size (nm)	Model Studied	Mechanism of Targeting/Delivery	Outcomes	Reference
Polymeric NPs	Poly (β-amino ester) NPs containing herpes simplex virus type I thymidine kinase	138 ± 4	In vitro: Rat glioma cell lines, 9L and F98 In vivo: 9L rat glioma model	Convection- enhanced delivery	100% cancer killing in in vitro, significant survival rate in rat models	[276]
Polymeric NPs	Bioreducible poly (β-amino ester) NPs containing miRNAs (miR-148a and miR296-5p)	100	In vitro: Human GBM cells	Direct intra-tumor infusion	Higher cellular uptake of miRs, efficient knockdown of Dnmt1 and Hmga1 (target genes of miRs), increased cytotoxicity	[277]
Albumin NPs	Paclitaxel-loaded human serum albumin NPs (HSA-PTX) and SP peptide anchored (SP-HSA-PTX)	HSA-PTX, 154.2 ± 12.6; SP-HSA-PTX, 168.2 ± 10.3	In vitro: Glioma U87 cells In vivo: Mice glioma model	SP neuropeptide specifically binds with NK-1 receptor facilitating BBB crossing and glioma targeting	Better stability and less drug leakage, increased drug uptake in SP-HSA-PTX, increased cytotoxicity, increased survival time and decreased tumor growth	[278]
Albumin NPs	Human serum albumin NPs encapsulating curcumin and coated with membrane of erythrocyte and DSPE-PEG3400-T807 (brain targeted ligand)	116.3 ± 0.8	Brain capillary endothelial cells, primary rat astrocyte cells and HT22 cells	Lipid insertion method	Improved affinity to neuronal cells, sustained release of curcumin until 72 h, no non-specific cytotoxicity	[279]
Gold NPs	Transferrin peptide targeted gold NPs (Tfpep-Au NPs) for delivering photosensitizer	Au NPs, 8.2, PEGylated Au NPs, 10.1 Tfpep-Au NPs, 12.3	Human glioma cell lines, U87 and LN229	TF binds to TF receptor followed by endocytosis and accumulation in mitochondria of tumor cells	Tfpep-Au NPs-Pc 4 elicited increased PDT cytotoxic efficacy than free PEGylated and Au PEGylated NPs	[280]
Gold NPs	Angiopep-2 decorated gold NPs encapsulating doxorubicin (An-PEG-DOX-AuNPs)	AuNPs, 25.01 ± 0.10 PEG-DOX-AuNPs, 35.97 ± 0.72 An-PEG-DOX-AuNPs, 39.96 ± 0.57	In vitro: CR glioma cells In vivo: Kumming mice having glioma	Incorporation of hydrazone, an acid-responsive linker facilitates the release of drug, and Angiopep-2 interacts with LRP1 receptor to enable the entry across the BBB	An-PEG-DOX-AuNPs elevated anti-glioma effects, increased survival rate of mice more than 2.8-fold	[281]
Magneto-electric NPs	Hydrothermally constructed magneto-electric NPs with CoFe_2_O_4_@BaTiO_3_ for delivering MIA690 (antagonist peptide for growth hormone-releasing hormone)	30	U-87 MG cells	Applied intravenously and directed under magnetic field	Increased specificity, improved cellular uptake, increased cytotoxicity	[282]
Magneto-electric NPs	Biocompatible magnetic iron oxide NPs with trimethoxysilylpropyl-ethylenediamine triacetic acid encapsulating doxorubicin (DOX-EDT-IONPs)	DOX-EDT-IONPs, 75.5 ± 3.2	bEnd.3, Madin–Darby canine kidney transfected with multi-drug resistant protein 1 (MDCK-MDR1) and human U251 GBM cells	Incorporation of external magnetic field and cadherin (ADTC5)-binding peptide promotes the BBB penetration	Suppresses U251 cell proliferation, increased doxorubicin uptake, increased TOP II and Ku70 (enzymes for DNA repair, replication) and reduced caspase 3, p53 expression	[283]
Quantum dots	Large amino acid-mimicking carbon quantum dots (LAAM CQDs) manufactured by mixing 4,5,8-tetraminoanthraquinone (TAAQ) and citric acid (CA)	-	Tumor-bearing female BALB/c mice	The presence of α-carboxyl and amino groups enables interaction with LAT1 and promotes drug delivery	Improved drug delivery and tumor imaging, decrease in tumor load	[284]
Quantum dots	Non-functionalized graphene quantum dot (NF-GQDs) and dimethylformamide-functionalized GQDs (DMF-GQDs)	<10	U87 human GBM and primary cortical neurons	Interaction of GQDs alters the membrane fluidity enabling delivery of negatively charged NPs	Significant cytotoxicity through delivery of doxorubicin via DMF-GQDs, biocompatible QDs	[226]
Upconversion NPs (UCNPs)	Assembly of oleic acid-coated UCNPs giopep-2/cholesterol-conjugated poly(ethylene glycol) and the hydrophobic photosensitizers (ANG-IMNPs)	74 ± 4	In vitro: ALTS1C1 astrocytoma cells In vivo: orthotropic tumor-bearing mice models	Targeted delivery through Angiopep-2	Enhanced uptake, significant cytotoxicity, selectively delivered dual photosensitizers for combined photothermal/photodynamic therapy, prolonged survival in vivo	[232]
Biomimetic NPs	Zoledronate encapsulated NPs coated with microglia cell membrane (ZOL@CNPs) Chemoattractant driven and microglia based	ZOL@NPs, 188 ZOL@CNPs, 204	GL261/TR cells, bEnd.3 cells for BBB model In vivo: TMZ-resistant GBM mice	Presence of glutathione-enhanced release of ZOL, high GSH concentration, interaction between chemoattractants (CX3CL1 and CSF-1) secreted by GL261/TR cells and its receptor (CX3CR1 and CSF-1R) on the surface of ZOL@CNPs promotes entry into BBB	Reduced tumor growth by inducing apoptosis, inhibiting the migration and invasion of resistant cells	[285]
Biomimetic NPs	Red blood cell membrane-coated solid lipid nanoparticle (RBCSLN)-based nanocarrier dual-modified with T7 and asparagine–glycine–arginine (NGR) peptide (T7/NGR-RBCSLNs) containing vincristine	123.67 ± 0.65	C6 cells (rat glioblastomas) Zebra fish	Interaction of peptides with receptors on membrane surface followed by internalization	Improved anti-glioma effect	[286]
Nucleic acid-NPs	Liposome NPs siRNAs/AntimiR-21 coupled with chlorotoxin	<180	In vitro: U87 human GBM and GL261 mouse glioma cells In vivo: Glioma mouse model	Interaction of CTX on liposome surface enables internalization	Increased levels of tumor suppressor proteins, activated caspases 3/7, reduced tumor growth	[287]
Nucleic acid-NPs	Polyethylenimine-coated spherical NPs encapsulating Gli 1, a TF in Hedgehog signaling pathway	~100	U87-MG	Binds to the scavenger receptors on glioblastoma cells (GBM) and undergoes endocytosis in a caveolae/lipid raft/dynamin-dependent way	Achieved silencing of tumor-promoting Hedgehog pathway genes, alterations of proteins for chemoresistance of GBM, suppression of stemness genes and reduced self-renewal capacity, improved neurosphere chemosensitivity	[288]
Exosomes	Neutrophil exosomes loaded with doxorubicin	112.5 ± 12.6	In vitro: bEnd.3 (Mouse brain endothelial cells) and C6 In vivo: Zebrafish and C6-Luc glioma-bearing mice models	Internalization through clathrin endocytosis and glioma targeted delivery	Specific activity towards brain inflammation, significant anti-glioma effect	[289]
Exosomes	Brain endothelial-derived exosomes encapsulating vascular endothelial growth factor (VEGF) siRNAs developed using cationic liposomal transfection	-	In vitro: bEnd.3 In vivo: Zebrafish as xenograft brain cancer model	Tetraspanins, such as CD63 favors cell–cell communication and directs the entry to BBB	Improved uptake of siRNA Silencing of VEGF required for tumor progression	[266]
